# Inter-provincial disparity of COVID-19 transmission and control in Nepal

**DOI:** 10.1038/s41598-021-92253-5

**Published:** 2021-06-25

**Authors:** Buddhi Pantha, Subas Acharya, Hem Raj Joshi, Naveen K. Vaidya

**Affiliations:** 1 Department of Science and Mathematics, Abraham Baldwin Agricultural College, Tifton, GA 31793 USA; 2 Department of Mathematical Sciences, University of Texas at Dallas, Dallas, TX USA; 3 Department of Mathematics, Xavier University, Cincinnati, OH USA; 4 Department of Mathematics and Statistics, San Diego State University, San Diego, CA USA; 5 Computational Science Research Center, San Diego State University, San Diego, CA USA; 6 Viral Information Institute, San Diego State University, San Diego, CA USA

**Keywords:** Infectious diseases, Mathematics and computing

## Abstract

Despite the global efforts to mitigate the ongoing COVID-19 pandemic, the disease transmission and the effective controls still remain uncertain as the outcome of the epidemic varies from place to place. In this regard, the province-wise data from Nepal provides a unique opportunity to study the effective control strategies. This is because (a) some provinces of Nepal share an open-border with India, resulting in a significantly high inflow of COVID-19 cases from India; (b) despite the inflow of a considerable number of cases, the local spread was quite controlled until mid-June of 2020, presumably due to control policies implemented; and (c) the relaxation of policies caused a rapid surge of the COVID-19 cases, providing a multi-phasic trend of disease dynamics. In this study, we used this unique data set to explore the inter-provincial disparities of the important indicators, such as epidemic trend, epidemic growth rate, and reproduction numbers. Furthermore, we extended our analysis to identify prevention and control policies that are effective in altering these indicators. Our analysis identified a noticeable inter-province variation in the epidemic trend (3 per day to 104 per day linear increase during third surge period), the median daily growth rate (1 to 4% per day exponential growth), the basic reproduction number (0.71 to 1.21), and the effective reproduction number (maximum values ranging from 1.20 to 2.86). Importantly, results from our modeling show that the type and number of control strategies that are effective in altering the indicators vary among provinces, underscoring the need for province-focused strategies along with the national-level strategy in order to ensure the control of a local spread.

## Introduction

**Figure 1:**
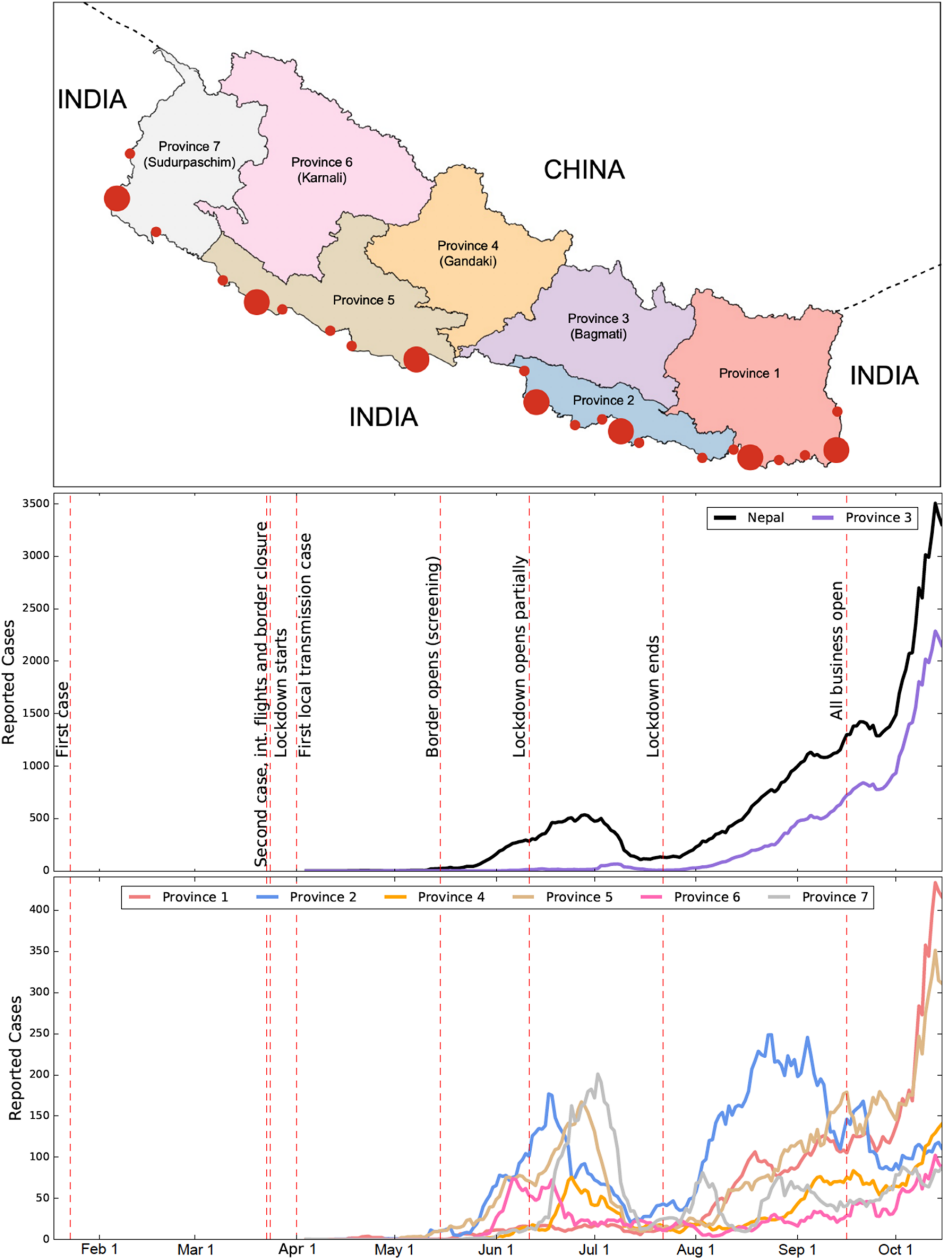
(Top Panel) A map of Nepal showing its seven provinces and entry-points (filled red circles) along the border with India. To create the map, the data (shapefile format) was obtained from the official webpage (http://dos.gov.np) of the government of Nepal (Accessed on April 23, 2021)^45^. The map was then created using GeoPandas Verson 0.8.1 (https://geopandas.org/index.html)^45^ module in Python programming. The large-sized circles indicate the major entry points: Kanchanpur, Nepalganj, Siddharthanagar, Birgunj, Jaleshwar, Biratnagar, and Kakarbhitta (listed from the left to right). The small-sized circles indicate the minor entry points (official border crossings)^46^. (Middle and Bottom Panels) The number of reported cases of COVID-19 in Nepal and in its provinces from January 23, 2020 to October 15, 2020. Because of the high magnitude of the cases of Province 3, the daily new cases data are shown in the same graph (middle panel) for Province 3 and the whole country, while the daily new cases data for the remaining six provinces are shown together in a single graph (bottom panel). The vertical red dashed lines represent the dates corresponding to the policy changes by the government.

The COVID-19 disease, caused by the severe acute respiratory syndrome coronavirus-2 (SARS-CoV-2), has triggered devastating impacts worldwide, with more than 146 million cases and 3.1 million deaths as of April 24, 2021^1–7^. Since the first case reported in 2019, various efforts to contain the COVID-19 spread have been made all over the world^2,3,8,9^. Even though several vaccines have been developed, their proper implementation is expected to take a few more years, especially in developing countries. Moreover, the emergence of the number of novel SARS-CoV-2 strains has posed further threats to this pandemic^10^. Therefore, the non-pharmaceutical intervention still remains the primary means of fighting against this disease. In these control policy efforts, even identical non-pharmaceutical intervention policies have resulted in different epidemiological outcomes in different places^6,11–18^, indicating uncertainty in the pattern of the disease transmission, human behavior, and effective control strategies^18–22^. Therefore, studies focused on local-level transmission are necessary for identifying the control strategies that are suitable to mitigate the burdens of the outbreak in the specific region. In this study, we consider data related to the COVID-19 outbreak in seven provinces of Nepal (Fig. 1) to explore the province-level disease transmission trends and inter-provincial disparity of the effects of control strategies.

While the seven provinces of Nepal are interlinked with thousands of people traveling daily between the provinces for various reasons such as work, tourism, religion, health care, and business, population contact patterns within these provinces are quite different from each other. For example, Province 3 includes the capital city of the country with the highest population density, giving a dense contact pattern, and is the only province having the land and air traffic networks to all other provinces^23^. The Northern part of all provinces (except Province 2) have remote mountainous areas with low population densities, giving less dense contact patterns^24^. Some provinces (for example, Province 4) have a greater inflow of tourists compared to other provinces. Therefore, life-style, cultural activities, economic conditions, and urbanization result in quite distinct population contact patterns among these provinces.

More importantly, Nepal has a unique geographical condition due to its open-borders with some provinces with India, one of the most COVID-19 affected countries in the world (Fig. 1)^23,25,26^. The open-border provision further extends the contacts of people living in the border provinces to neighboring local communities of India, especially through common activities due to similar languages, writing-scripts, movies, musics, marriage ceremonies, and religious gatherings^24,27,28^. Because of the open-border relationship with India and job opportunities available in the major Indian cities, about 4 million Nepalese seasonal migrant workers work in India^26,29^. Due to the implementation of lockdown in India, tens of thousands of Nepalese migrant workers returned home in March and April of 2020, allowing a significant number of COVID-19 cases to enter Nepal^27,30–32^. Despite a significant inflow of cases through the Indian border, timely implementation of policies by the Nepal government, such as border screening followed by quarantine, countrywide lockdown, and isolation of infected individuals, was successful in controlling the local spread of the disease until June 11, 2020 (less than 900 reported local transmission out of 4,300 total number of cases)^24,33^. When the policies were lifted phase-wise beginning on June 11, 2020, the COVID-19 cases surged uncontrollably^1,30^, indicating that the previously implemented policies were quite successful. Thus, the unique data set from Nepal with a multi-phasic trend of disease dynamics allows us to gain insights into the COVID-19 transmission dynamics and effective control strategies in the context of geographic and demographic heterogeneity that exists among provinces of Nepal.

Several modeling techniques have been widely applied to study the different aspects of the COVID-19 pandemic and evaluate the impacts of non-pharmaceutical interventions on controlling its transmission. Statistical techniques, such as reduced form econometric method^34^ and regression analysis^4^, have been used to evaluate control measures. Some studies have provided estimates of the reproduction number using data from several places^14,35–38^ and have observed its link to prevention strategies. Many variants of deterministic and stochastic models have also been developed^4,6,14,17,18,33,35,36,39–43^ to predict COVID-19 transmission dynamics, estimate basic reproduction numbers, and examine intervention strategies such as travel ban, lockdown, isolation, and quarantine. While the previous studies have provided important insights into the on-going pandemic, none of these studies focused on epidemic indicators in the context of geographical (open-border) and population contact disparity that exists in places like the provinces of Nepal.

In this study, we compile and analyze province-wise data from Nepal on reported cases, active cases, testing, isolation, and quarantine^1,23^. We then use the compiled data to study how heterogeneity among provinces can impact the outbreak indicators, such as epidemic trend, epidemic growth rate, and reproduction numbers. Furthermore, we implement modeling via multiple linear regressions to identify the control policies that were effective in altering these indicators in each province. The primary control strategies implemented by the government of Nepal that we consider in this study are lockdown, testing, isolation of positive cases, and quarantine for the people entering from the border. We also compare province-wise results with the results for the whole country to study how the national-level policies get translated to the province-level controls.

## Results

### Epidemic trend

We present the general trend of the COVID-19 outbreak in Nepal and in its provinces using the compiled data (Fig. 1). In particular, we focused on the timing of surges in COVID-19 cases and the early linear growth rates of those surges. Despite the first COVID-19 case confirmed on January 23, 2020 and the second case detected on March 24, 2020 the cases remain negligible until May 20, 2020 due to timely implementation of interventions by the government of Nepal (March 24, 2020: closure of international flights, border closure, and the start of lockdown)^30,33,44^. The data shows that the country has faced a multi-phasic epidemic pattern with three surge periods of distinct magnitude: low (May 20 to June 25, 2020), medium (July 22 to September 20, 2020), and high (post-September 16, 2020). The timing for the beginning of these surges approximately corresponds to the border opening ($\sim$ May 20, 2020), lockdown ending ($\sim$ July 21, 2020), and countrywide travel opening ($\sim$ September 20, 2020), respectively. Modeling the beginning of these surges by the linear growth function, we found that the average number of new infections grew linearly with a slope 15 per day, 24 per day, and 154 per day during the low, medium, and high surge periods, respectively (Table 1). A dramatic increase in the cases during the high surge period implies a significant impact of policies that were implemented prior to September 16, 2020 when all the policies were lifted.

We note that the first low-level surge can be attributable to the inflow of COVID-19 cases from India. In mid-May of 2020, the government opened the southern border partially to let seasonal labor migrants return home. They were screened and quarantined, and were isolated if tested positive. The inflow of the positive cases through the Indian border resulted in the first surge leading to as high as 671 cases per day (June 18, 2020). Because of border screening, quarantine, testing, and isolation, the potential local transmission was avoided, and the case number declined to about 100 per day by mid-July of 2020. However, after the Nepal government ended the lockdown policy on July 21, 2020, the second surge began primarily due to the community transmission, leading up to 1,460 new cases per day on September 15, 2020. Moreover, after the government resumed all activities on September 16, 2020, the third surge began, leading to 3,750 new cases per day on October 15, 2020.

**Table 1:** The slope of the linear model and the duration of the linear trend for three surge periods.

Regions	Nepal	Province 1	Province 2	Province 3	Province 4	Province 5	Province 6	Province 7
First surge period	5/20–6/ 25	5/15–6/15	5/10–6/18	6/01–7/08	6/05–6/24	5/12–6/27	5/22–6/06	6/01–7/01
Slope	14.8	0.6	5.1	0.6	4.2	3.6	7.12	8.2
Second surge period	7/22–9/20	7/16–8/18	7/22–8/24	8/01–9/25	8/10–9/10	8/03–9/16	8/10–9/18	8/15—8/27
Slope	23.4	3.2	7.1	16.8	2.5	3.09	0.7	5.3
Third surge period	9/26–10/15	9/25–10/13	9/29–10/15	9/27–10/13	9/29–10/15	10/01–10/13	9/29–10/13	9/24–10/03
Slope	153.3	21.0	2.6	103.9	6.3	19.0	5.3	5.5

We also analyzed the province wise data to observe any disparities in the epidemic trend of COVID-19 among seven provinces. While the first surge of new cases began in all provinces after the government opened the Indian border (May 20, 2020), the subsequent surge patterns were quite different among the provinces, in both peak timing and magnitude (Fig. 1). Our analysis using linear model approximation on three surges (Table 1) shows that the different provinces have different time intervals during which the linear growth was observed, and the slope of these linear growths varies widely among provinces. Province 7 had the first linear growth pattern (June 1–July 1, 2020) with the highest slope (8 new cases per day), while Province 5 has the longest interval (May 12–June 27, 2020) with a slope of 3.6. Similarly, Province 2 and 5 have the earliest linear growth pattern starting on May 10 and 12, 2020, and ending on June 18 and 27, 2020, respectively. In general, the provinces with a higher inflow of migrant workers from India (Province 2, 6, 7) have a higher slope of the linear growth rate during first surge period, while the provinces with a lower inflow of migrant workers from India (Province 1 and 3) have higher slope during the later surge period, which occurred after the government started to lift the restraining policies. Note that Province 3, which does not have an Indian border entry point, has the smallest slope during the first surge period (June 01 to July 01, 2020). However, it has the highest slopes of 17 and 104 cases per day during the second (August 1 to September 25, 2020) and the third (September 27 to October 13, 2020) surge periods, respectively. These extremely high cases in Province 3, which contribute to about 2/3 of the total cases in the country during the third surge period, could be because this province includes the capital city of Nepal, making it the highest populated province with the maximum population contact network. Overall, our analysis on the disease transmission trend identifies that both geographical situation (mobility across the border with India), as well as internal demography (population size) and lifestyle (contact network), contribute to the provincial disparities on the epidemic trend of COVID-19 in Nepal.

### Daily exponential growth rate

As revealed in the disease epidemic patterns (Fig. 1), while the linear growth can be observed for a short time, the disease pattern was nonlinear, indicating the time-dependency of the rate in which the number of cases changes. To better describe the time-dependent growth pattern, here we compute the daily exponential growth rates following the techniques implemented in Hsiang et al.^34^. Such techniques, often used in economic growth^47,48^, were also proved to be extremely useful in many diseases, such as COVID-19^34^ and influenza^49^, to analyze time-varying epidemic growth rate that can be affected by policies and other conditions.

**Figure 2:**
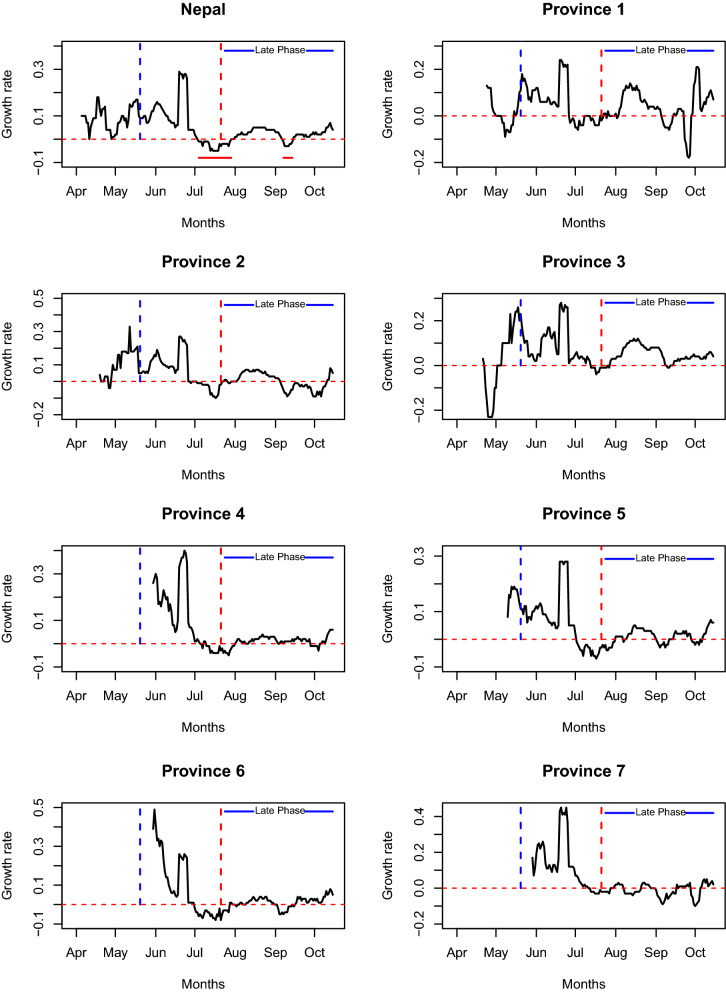
Time-dependent daily exponential growth rate of COVID-19 in Nepal and in its provinces during 2020 pandemics. The vertical blue dotted lines indicate the start date of border opening with screening and the vertical red dotted lines indicate the date at which the countrywide lockdown was ended, after which the epidemic is described as the late phase (solid blue line). The horizontal red line segments in the first graph indicate the time interval at which the countrywide epidemic growth rates are negative. Such line segments are not indicated in the graphs of provinces due to frequently alternating pattern of positive and negative growth rates in individual province. The horizontal red dotted lines indicate the epidemic growth rate of 0.

**Figure 3:**
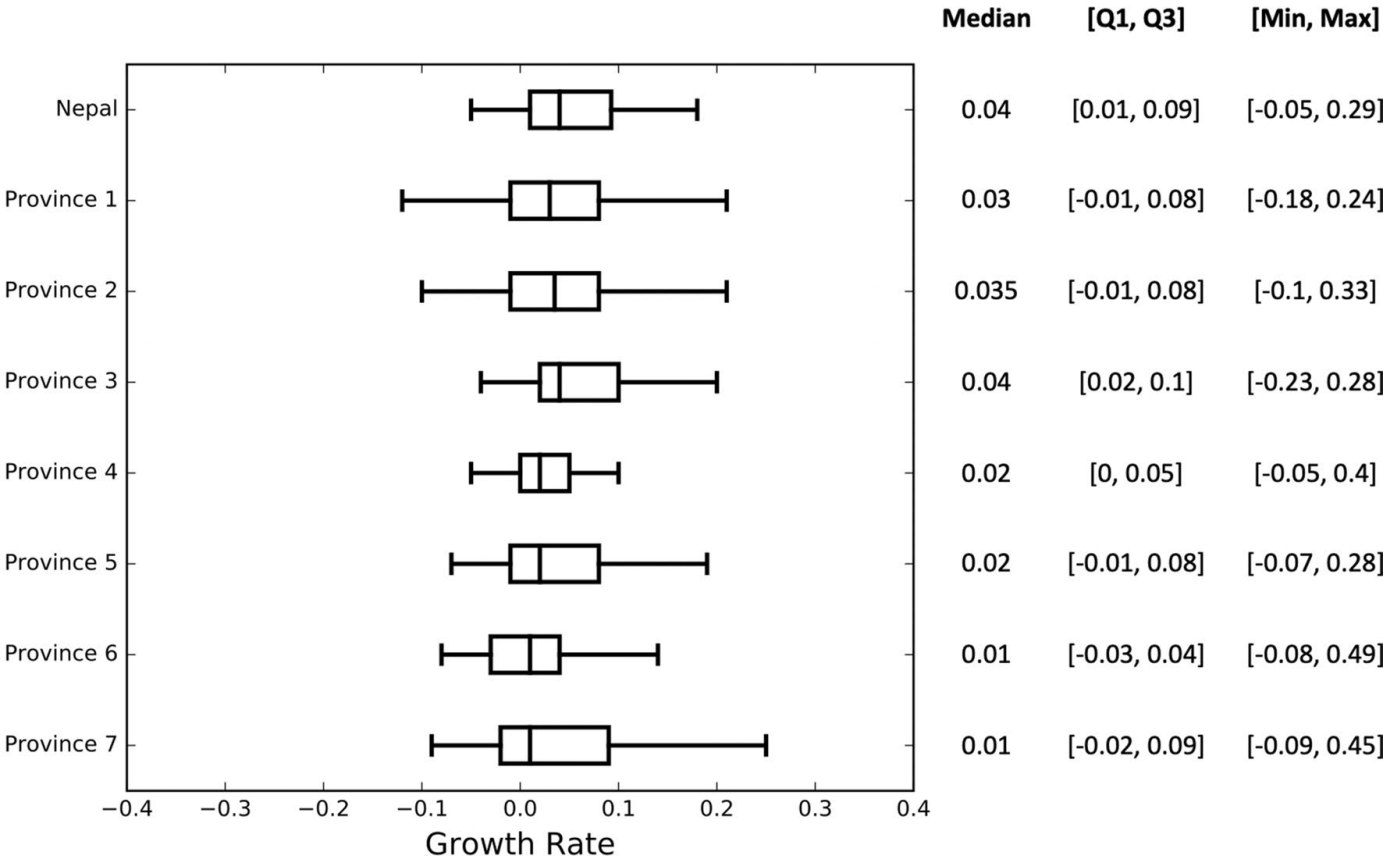
The five number summary (median, lower quartile, upper quartile, minimum, maximum) and the corresponding box plots of the daily exponential growth rates in Nepal and its provinces during the entire epidemic period considered.

Our computations clearly show the temporal change of the epidemic growth rate (Fig. 2), which is also supported by the significantly large inter-quartile range (Fig. 3). Throughout the study period from April 4 to October 15, 2020, the country-wide daily growth rate was almost always positive except in two short intervals from July 4 to 28, 2020 and from September 7 to 14, 2020 (indicated by horizontal solid red line segments in the first plot of Fig. 2). We observed the sudden increase in the daily growth rate (from negative to positive values) during the end of July and the middle of September, 2020, which may be attributable to the major alteration of the policies, such as the lifting of the lockdown (July 21, 2020) and the opening of all business, including international flights (September 16, 2020), respectively. Furthermore, a drastic rise in the growth rate occurred following the relaxation of the phase-wise lockdown (June 18, 2020). During the study period, the number of active cases was changing with the median exponential growth rate of 4% per day (Quartile: [1%, 9%] per day; min-max: [− 5%, 29%] per day) (Fig. 3).

For each province, we observed the median value of the growth rate to be positive (1–4%), while the magnitude varied dynamically between negative and positive values throughout the study period (Fig. 3). Comparison of the growth rates among provinces reveals that there are some noticeable differences in the growth pattern. In Province 1, 2, 3, and 5, the epidemic growth began before the border opening with screening on May 20, 2020 (blue vertical lines in Fig. 3), but other provinces experienced the epidemic growth only after May 20, 2020. Also, unlike other provinces, Province 3 had a negative growth rate at the beginning of the pandemic (April 23–May 5, 2020), and then the growth rate remained positive almost all the time period considered except during a brief interval (July 13–25, 2020) ( Fig. 2). The absence of inflow of migrants through the border with India and controlled local transmission due to lockdown policy may have contributed to the negative growth rate of Province 3 during the early phase. However, Province 3 also suffered from one of the highest growth rates during the later part of the epidemic as shown in Figs. 2 and  3 (median growth: 4% per day; quartiles [$Q_{1}=2\%, Q_{3}=10\%$]). Immediately after the border opening, the daily growth rate increased in the provinces having a higher inflow of migrants from India (Province 1, 2, 5, 7).

### Reproduction numbers

While the growth rate computed above is useful to observe the day-to-day microscopic trend of the epidemics, it may not be a good indicator to capture the general trend of the disease due to its inability to include the disease-state specific distribution of infectiousness. The general trends can be better described by the reproduction number of the disease^50–53^. Here we utilize the compiled data to estimate the basic reproduction number and the effective reproduction number for provinces as well as for the whole country.

#### Basic reproduction number, $R_{0}$

The basic reproduction number is defined as the number of secondary infections produced by an infected individual in a pool of a completely susceptible population during its infectious period. In general, if the value of the basic reproduction number is greater than unity, then the disease spreads, and if the value is less than unity, the disease dies out^50–52^. In Table 2, we present the values of basic reproduction number along with their 95% confidence intervals for Nepal and its provinces. We estimated the basic reproduction number for the whole country to be 1.083 (95% CI 1.075–1.093 ) (Table 2), which is comparable to $R_{0}$ estimated for other countries^33,36,39,40,43,54,55^.

**Table 2:** The basic reproduction number, $R_{0}$, of COVID-19 for Nepal and its seven provinces. The third column shows the 95% confidence interval of the estimated values of $R_{0}$.

Province	$R_{0}$ Value	95% CI
Nepal	1.083	[1.075, 1.093]
Province 1	1.137	[1.107, 1.166]
Province 2	0.835	[0.815, 0.855]
Province 3	1.205	[1.191, 1.219]
Province 4	1.047	[1.009, 1.085]
Province 5	0.853	[0.831, 0.875]
Province 6	0.707	[0.672, 0.743]
Province 7	0.977	[0.947, 1.008]

Among the seven provinces, the highest reproduction number was estimated in Province 3 ($R_{0}=1.205$, 95% CI: 1.191–1.219 ) followed by Province 1 ($R_{0}=1.137$, 95% CI: 1.107–1.166 ) and then by Province 4 ($R_{0}=1.047$, 95% CI: 1.009–1.085 ). Since Province 1, 3, and 4 are the ones with the highest populous cities, the higher likelihood of contacts may have contributed to their higher values of $R_{0}$. Note that the other provinces (2, 4, 5, 6) have values of $R_{0}$ less than 1 (Table 2), indicating the local spread of the disease in these provinces was under control during the initial phase. The observed disparity of $R_{0}$, not only on its magnitude but also on its level to be less or greater than unity, highlights that the province-level $R_{0}$ may be necessary to accurately predict whether a province-wise outbreak occurs or not. It’s worth noting that while some provinces had the epidemic under control ($R_{0}<1$) because of the strict on-time government policies, the disease eventually spread to those provinces once the control policies were lifted, as indicated by the data.

#### Effective reproduction number, $R_{t}$

**Figure 4:**
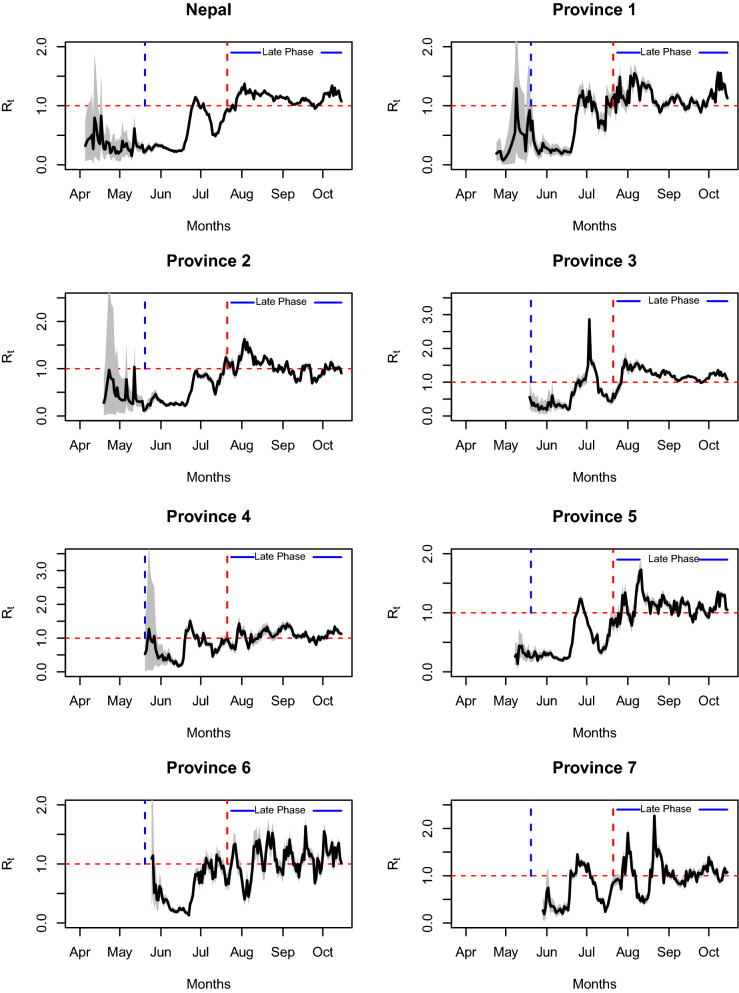
The time-dependent effective reproduction number of COVID-19 for Nepal and its seven provinces during 2020 pandemic. The gray shaded region is the 95% confidence interval for $R_{t}$. The solid curve in the middle of the gray area is the average effective reproduction number. The vertical blue dotted lines indicate the start date of border opening with screening and the vertical red dotted lines indicate the date at which the countrywide lockdown was ended, after which the epidemic is described as the late phase (solid blue line). The horizontal red dotted lines indicate the effective reproduction number of 1.

One of the main assumptions while calculating the basic reproduction number, $R_{0}$, is that all individuals who are not infected are equally susceptible to the virus. While this is usually true at the beginning of an epidemic, the susceptible populations change over time as some individuals in the population may be recovered or immune to the disease or become protected due to intervention policies. This implies that the actual number of secondary infections per infected individual changes over time, partly due to the impact of the policy changes. The number of secondary cases per infected individual in a population made up of both susceptible and non-susceptible individuals is known as the effective reproduction number (denoted by $R_{t}$)^56^. In general, $R_{t} < 1$ ($R_{t} > 1$) indicates that the epidemic trend at time *t* is decreasing (increasing). Our estimated profile of the effective reproduction number in Nepal and its provinces, along with their 95% Credible Interval (gray region), is presented in Fig. 4. Note that as required by our method^57^ we started computing $R_{t}$ only after the time point when there are at least some consecutive non-zero incidences and no sequence of consecutive zeros afterward.

Our estimates show that the effective reproduction number of Nepal stayed below unity at almost all time points before the government started to ease the phase-wise lockdown (on June 11, 2020), indicating a decreasing trend in this period, consistent with the controlled phase of the epidemic. The observed decreasing trend ($R_{t}<1$) for the initial phase, despite the initial surge of COVID-19 cases, is consistent with the fact that the initial increase in cases is attributable to imported cases rather than local transmission. After June 11, 2020, the effective reproduction number exceeded 1 and reached 1.24 on June 23, 2020, and then remained above 1 for the next 2 weeks. After the end of lockdown (July 21, 2020), a further increase of $R_{t}$ took place, making it 1.40 on July 30, 2020, and then it stayed above 1 almost all the time throughout the study period. Note that the pattern of outgrowing cases after the relaxation of policies is well represented by our estimates of $R_{t}$. The trend of $R_{t}$ observed in our study is consistent with the trend observed in a previous differential equation modeling study^33^, with our estimates being slightly smaller than the estimates from the differential equation modeling, similar to the finding in other places^35^. This discrepancy may be described by the fact that our data includes only the recorded cases, missing those asymptomatic cases which were not recorded.

The temporal pattern of the effective reproduction number in all provinces appeared to be more or less similar after July 21, 2020, showing that all provinces experienced the increasing trend of cases in the absence of full-fledged policies. However, the magnitude of $R_{t}$ varies from province to province. For example, in Province 3, the value of $R_{t}$ reached as high as 2.86 (July 3, 2020) and as low as 0.17 (June 1, 2020) with the highest variance ($s^{2}=0.20$), while Province 4 had the lowest variance ($s^{2}=0.09$) with the value of $R_{t}$ ranging from 0.16 to 1.5. More importantly, we observed that the pattern of $R_{t}$ is quite different among provinces while the policies were in place (before July 21, 2020). Because of the data having consecutive zeros at the beginning of pandemic in Province 3, 4, 6, and 7, the non-zero effective reproduction number appeared only after May 20, 2020 (blue vertical line in Fig. 4) when the government opened the border with screening policy, while Province 1, 2, and 5 had continuous non-zero cases, and thus non-zero values of $R_{t}$ before May 20, 2020. Province 2 is the first province that had the earliest value of $R_{t}$ greater than 1 ($R_{t}$ = 1.04 on May 12, 2020), while Province 3 had the value of $R_{t}$ greater than 1 only after more than a month, on June 25, 2020 ($R_{t}=1.07$), which corresponds to the timing of the relaxation of within province restriction. Also, Province 5 and 7 did not experience $R_{t} > 1$ (increasing trend) until July 5 and June 25, 2020, respectively. Moreover, right after June 22, 2020, Province 3 experienced a dramatic spike of $R_{t}$ leading to its value of 2.86 on July 3, 2020, while most of the other provinces had the value of $R_{t}$ less than 2 during the entire study period. These results show that the effectiveness of policies might vary across the provinces even though universal policies were implemented throughout the whole country.

### Impact of policies on the epidemic indicators

In this section, we used multiple linear regression models to study the effects of nation-wide implemented government policies on the control of COVID-19 spread. In particular, we estimated the potential impact of four government policies (predictor variables), lockdown, testing, isolation, and quarantine, on each of the three epidemic indicators (response variables), number of reported cases, the daily growth rate, and effective reproduction number. For the whole country as well as each province and for each epidemic indicator, we considered all possible models with one, two, three, and four predictor variables taken at a time. Then in each case, we compared the models using Akaike Information Criteria (AIC) to identify the best models that describe the data. The lower the AIC value, the better the model to describe the data. For the best models identified, we also provided the regression coefficients to quantify the impact of the policies in epidemic indicators.

#### Identification of the best models

The models having the lowest AIC values for 1, 2, 3, and 4 policies taken at a time are presented in Table 3. Our analysis of the whole country’s data indicates that each of the epidemic indicators is best described by the model containing all four policies. This shows that each of the policies implemented by the Nepal government had contributed to the successful control of the reported cases, daily growth rate, and effective reproduction number. To further analyze, we also observed the models restricted to 1, 2, and 3 policies. Interestingly, our model analysis identified that the policies that best describe epidemic indicators are not identical for all of these indicators. For example, in the case of one policy, the reported case or the growth rate as a response variable can be best described by the model with the isolation policy, while the effective reproduction number as a response variable can be best described by the lockdown policy. Similarly, for the models restricted to two policies, (testing, isolation), (testing, quarantine), and (lockdown, testing) were found to be the best predictors of reported case number, daily growth rate, and the effective reproduction number, respectively. Also, for the three-policy models, lockdown, testing, and isolation can best predict the reported cases and the effective reproduction number, while the daily growth rate can be best predicted by testing, isolation, and quarantine.

**Table 3:** The models having the lowest AIC values for three response variables (the reported cases, the daily growth rate, and the basic reproduction number), with four predictors (L-lockdown, T-testing, I-isolation, and Q- quarantine) taken 1, 2, 3, and 4 at a time. The bold-face indicates the best model identified according to AIC values for each province and the whole country.

	Reported cases		Growth rate		$R_{t}$	
	Predictors	AIC	predictors	AIC	Predictors	AIC
Nepal	I	2550	I	− 502	L	− 29.41
	T, I	2527	T, Q	− 529.21	L,T	− 111.11
	L, T, I	2481	T, I, Q	− 550.9	L, T, I	− 166.41
	**L, T, I, Q**	**2458**	**L, T, I, Q**	− **553.84**	**L, T, I, Q**	− **184.97**
Province 1	L	1755	I	− 374.6	T	62.98
	L, I	1713	I, Q	− 374.9	L, T	42.25
	L, T, I	1695	**L, T, Q**	− **379.8**	T, I, Q	33.25
	**L, T, I, Q**	**1691**	L, T, I, Q	− 378.4	**L, T, I, Q**	**30**.**86**
Province 2	L	2083	I	− 392.1	L	13.37
	L, Q	2051	I, Q	− 394.3	L, T	− 43.8
	**L, T, Q**	**2038**	**T, I, Q**	− **396.4**	**L, T, Q**	− **49.07**
	L, T, I, Q	2039	L, T, I, Q	− 394.8	L, T, I, Q	− 47.87
Province 3	I	2351	Q	− 312.4	L	117.6
	**L, I**	**2248**	T, Q	− 324.6	**L, Q**	**101**.**7**
	L, I, Q	2250	**T, I, Q**	− **328**	L, I, Q	102.4
	L, T, I, Q	2250	L, T, I, Q	− 327.0	L, T, I, Q	101.7
Province 4	Q	1685	L	− 231.3	**L**	**29**.**79**
	I, Q	1648	L, Q	− 239.6	L, Q	31.36
	**T, I, Q**	**1646**	**L, I, Q**	− **248.4**	L, I, Q	33.29
	L, T, I, Q	1648	L, T, I, Q	− 248.4	L, T, I, Q	34.90
Province 5	I	1922	T	− 351.5	L	6.611
	I, Q	1907	T, Q	− 400.6	L, T	− 16.33
	L, I, Q	1897	**T, I, Q**	− **405.7**	**L, T, I**	− **49.15**
	**L, T, I, Q**	**1880**	L, T, I, Q	− 405.5	L, T, I, Q	− 47.16
Province 6	Q	1670	Q	− 264.4	Q	13.2
	I, Q	1630	**T, Q**	− **273.3**	L, Q	8.619
	L, I, Q	1628	T, I, Q	− 272.2	**L, I, Q**	**7**.**757**
	**L, T, I, Q**	**1626**	L, T, I, Q	− 271.5	L, T, I, Q	9.722
Province 7	I	1969	Q	− 243.3	L	95.66
	I, Q	1963	I, Q	− 251.8	L, I	84.98
	T, I, Q	1958	**L, T, Q**	− **261**	L, T, I	83.88
	**L, T, I, Q**	**1956**	L, T, I, Q	− 259.9	**L, T, I, Q**	**83**.**32**

We performed a comparative analysis on the modeling of province-wise data to examine whether similar policies can predict the epidemic indicators at the province level. Our results show that the number of policies that predict the epidemic indicators are quite different at the province-level from those at the national level (Table 3). For example, except the reported case in Province 1, 5, 6, and 7 and the effective reproduction number in Province 1 and 7, all other indicators in all provinces are described by the model with less than four policies. For Province 2 and 4, the reported cases are predicted by three policies, while only two policies predict the reported case of Province 3. Similarly, three policies are required to best predict the daily growth rate of Province 1, 2, 3, 4, 5, and 7, and the effective reproduction number of Province 2, 5, and 6. The daily growth rate in Province 6 and the reproduction number in Province 3 can be best predicted by the model with two policies. Interestingly enough, only one policy (lockdown) was found to be the best predictor of the reproduction number in Province 4.

Not only the number of policies but also the type of policies as best predictors vary among provinces as well as among epidemic indicators. For example, even though the effective reproduction number is predicted by the same number of policies (3 policies) in Province 2, 5, and 6, the actual policies as predictors are (lockdown, testing, quarantine), (lockdown, testing, isolation) and (lockdown, isolation, quarantine) for Province 2, 5, and 6, respectively. Similar disparities were observed for the reported cases and the growth rates, as presented in Table 3. Such disparities among provinces as well as among indicators can be attributable to the province-wise heterogeneity in inflow of migrants through the border with India and the population contact network.

#### Regression coefficients of the best models

In Table 4, we present the regression coefficients, along with their 95% confidence interval, of the best models identified above for each of the three epidemic indicators. Here $\beta _{0}$ indicates the value of the epidemic indicators in the absence of policies, while the coefficients $\beta _{L}$, $\beta _{T}$, $\beta _{I}$, and $\beta _{Q}$ represent the slope (the rate of change of the indicator value per policy change) corresponding to lockdown, testing, isolation, and quarantine, respectively. We identified lockdown to be consistently effective to impact all indicators of the whole country, reducing the reported cases by 428 per day, the growth rate by 0.9%, and the effective reproductive number by 0.28. Surprisingly, our results provide positive coefficients for some indicators corresponding to some policies. While we acknowledge uncertainty on these coefficients due to misinformation in the data and the models, we note that these obscure findings can be specific to the context of Nepal. For example, an increase in the reported cases due to an increase in isolation and quarantine can be possible because the reported cases in Nepal at the beginning of the pandemic constitute those returning migrants who were quarantined and isolated following border screening. Also, an increase in the effective reproduction number due to an increase in testing or isolation could be because of the focus of testing and isolation on only those migrant workers screened at the border, who were less likely to be disease spreader in the local community. Further information, detailed data, and in-depth modeling can help ascertain these unclear results.

**Table 4:** Regression Coefficients of the best model for the reported cases, the daily growth rate and the effective reproduction number, $R_{t}$, in Nepal and its provinces.

Coefficients	$\beta _{0}$	$\beta _{L}$	$\beta _{T}$	$\beta _{I}$	$\beta _{Q}$
Region	**Reported Cases**				
Nepal	376.27 (273.37, 479.18)	-428.43 (-520.76, -336.10)	-0.0128 (-0.015, -0.010)	0.131 (0.114, 0.148)	0.0029 (0.002, 0.004)
Province 1	42.6580 (26.081, 59.235)	-34.2663 (-48.027, -20.505)	0.0194 (0.012, 0.027)	0.0863 (0.057, 0.126)	-0.011 (-0.020, -0.002)
Province 2	114.7375 (93.179, 136.296)	-123.1147 (-144.688, -101.541)	-0.0078 (-0.012, -0.004)	-	0.0115 (0.008, 0.015)
Province 3	210.7311 (178.294, 243.168)	-222.6894 (-260.001, -185.377)		0.3595 ( 0.320, 0.399)	
Province 4	-13.9620 (-23.237, -4.687)		0.0437 ( 0.000 0.087)	0.0503 (0.035, 0.066)	0.0121 (0.006, 0.018)
Province 5	30.7291 (11.365, 50.093)	-53.4800 (-72.147, -34.813)	-0.0038 (-0.005, -0.002)	0.0798 (0.065 , 0.094)	0.0029 (0.002 , 0.004)
Province 6	5.1897 (-3.371, 13.751)	-10.1369 ( -18.158, -2.116)	-0.0026 (-0.005, -3.61e-05p)	0.0688 (0.042, 0.095)	0.0033 (0.003, 0.004)
Province 7	-9.3865 (-27.942 , 9.169)	19.5248 (0.093, 38.957)	-0.0339 (-0.055, -0.013)	0.0504 (0.033, 0.068)	0.0007 ( 6.75e-05, 0.001)
	**Growth Rate**				
Nepal	0.0826 ( 0.053, 0.1120	-0.0090 (-0.035, 0.017)	-2.172e-06 (-2.92E-06, -1.42e-06)	-9.502e-06 (-1.44e-05, -4.64e-06)	9.477e-07 (6.24e-07, 1.27e-06)
Province 1	-0.0557 ( -0.118, 0.007)	-0.0685 (-0.113, -0.024)	-3.036e-05 (-5.18e-05, -8.96e-06)		9.847e-05 (6.03e-05, 0)
Province 2	0.0828 (0.056, 0.109)		-4.601e-06 ( -9.12e-06, -8.01e-08)	-5.882e-05 (-7.77e-05, -4e-05)	5.502e-06 (1.72e-06, 9.28e-06)
Province 3	0.0125 (-0.026, 0.051)		-9.507e-06 (-1.39e-05, -5.11e-06)	-3.467e-05 (-6.42e-05, -5.17e-06)	7.912e-0 (4.19e-05 0.000)
Province 4	0.3820 (0.222, 0.542)	0.1254 ( 0.090, 0.161)		0.0001 (4.55e-05, 0.000)	-0.0002 (-0.0003,-0.0001)
Province 5	0.0701 (0.034, 0.106)		( -1.24e-05, -8.18e-06) -1.029e-05	-3.144e-05 (-5.49e-05, -7.99e-06)	3.623e-06 (2.41e-06, 4.83e-06)
Province 6	0.0099 (-0.027 , 0.047)		-2.162e-05 (-3.45e-05, -8.77e-06)		1.544e-05 (1.27e-05, 1.82e-05)
Province 7	-0.0297 (-0.064, 0.005)	0.0767 ( 0.036, 0.117)	-3.93e-05 (-6.14e-05, -1.72e-05)		4.208e-06 (2.59e-06, 5.83e-06)
	**Effective Reproduction Number** $R_{t}$				
Nepal	0.6356 (0.561, 0.710)	-0.2894 (-0.355, -0.223)	1.123e-05 (9.32e-06, 1.31e-05)	5.583e-05 (4.34e-05, 6.82e-05)	-1.923e-06 (-2.75e-06, -1.1e-06)
Province 1	1.2529 (0.905, 1.67)	-0.1808 (-0.353, -0.009)	0.0003 ( 0.0002, 0.0004)	-0.0009 (-0.001, -0.0008)	-0.0004 (-0.001, -0.0002)
Province 2	0.8816 (0.808, 0.955)	-0.4211 (-0.495, -0.347)	5.083e-05 (3.8e-05, 6.36e-05)		-1.537e-05 (-2.66e-05, -4.1e-06)
Province 3	0.7627 ( 0.538, 0.987)	-0.5307 (-0.653, -0.408)			0.0004 (0.000, 0.001)
Province 4	1.0735 (1.009, 1.138)	-0.3445 (-0.438, -0.251)			
Province 5	0.6703 (0.554, 0.787)	-0.4866 (-0.564, -0.409)	3.182e-05 (2.38e-05, 3.99e-05)	0.0003 (0.0002,0.0004)	
Province 6	1.2430 (1.113, 1.373)	-0.1413 (-0.257, -0.025)		-0.0004 (-0.001, 6.66e-05)	-4.739e-05 (-5.95e-05, -3.53e-05)
Province 7	0.8505 ( 0.676, 1.025)	-0.2037 ( -0.373, -0.035)	-0.0001 (-0.000 , 1.36e-05)	0.0002 (0.0001, 0.0003)	-5.261e-06 (-1.19e-05, 1.36e-06)

We also provided the coefficients for the best models identified for each province (Table 4). A widely varying magnitude of estimated coefficients implies that the net effects of policies on the epidemic indicators vary among provinces. For example, lockdown was found to reduce the effective reproduction number from the minimum of 0.14 in Province 6 ($\beta _{L}=-0.14$) to the maximum of 0.53 in Province 3 ($\beta _{L}=-0.53$) . Surprisingly, the impact of the same policy on the same epidemic indicator was found to be positive in one province and negative in another province. While lockdown is mostly effective in reducing these indicators in most of the provinces (negative values of $\beta _{L}$), our modeling resulted in the positive value of $\beta _{L}$ for the reported cases in Province 7 and the daily growth rate in province 4 and 7. Even though these results appear to be paradoxical, it may be quite possible to be true in the context of Nepal due to geographical heterogeneity. In particular, Province 7’s border has one of the most in-and-out flows of migrants. As a result, the reported cases, as well as the growth rate, can increase due to inflow of cases from India even after lockdown is implemented.

## Discussion

Although multiple COVID-19 vaccines have recently been developed, proper implementation remains a major issue for many countries, especially developing countries like Nepal, where poor management and availability of resources are often obstacles in public health programs. Since the long-term efficacy of the vaccines are not well-known, and a considerable portion of the population may be reluctant to receive the vaccines during the early phase of vaccination, the disease spread has been expected to continue for a longer time period. Also, the emergence of new strains of SARS-CoV-2, such as UK, Brazil, and South African strains, has posed a continued threat of future COVID-19 surge. Therefore, non-pharmaceutical interventions are still the recommended strategies for the prevention of disease spread. The effectiveness of various non-pharmaceutical interventions on controlling the disease has not been fully understood, particularly in the context of local-level heterogeneity in terms of geographical background and population contact network. Here, we took advantage of unique province-wise data set from Nepal that allowed us to quantify the impact of national-level policies (lockdown, testing, isolation, quarantine) on the province-wise epidemic indicators (reported cases, daily growth rate, effective reproduction number).

Presumably because of unique geographical and demographic features, our analysis and modeling of data from Nepal revealed a number of interesting findings regarding COVID-19 transmission and control policies. For each of the widely used epidemic indicators (reported case number, daily growth rate, and effective reproduction number), both the geographical situation, such as the open border with India, and contact network, such as population size and lifestyle (urban or rural), could play significant role in causing province-wise disparity to these indicators. The movement of seasonal labor migrants across the Indian border was primarily responsible for the first surge of COVID-19 in Nepal. However, the peak of this initial surge and the slope of the early linear trend of reported cases remained quite low, highlighting the effectiveness of policies that the Nepal government implemented right at the beginning of the pandemic. The dramatic surge with a high slope of the linear trend after lifting the policies also asserts the successful impact of these policies in the context of Nepal, where mobility across the open border is a huge issue for disease control. While these policies were in place (i.e., until June 2020), the effective reproduction number remains quite low below 1 (decreasing trend of local transmission), which after the relaxation of policies frequently increases to above 1 (increasing trend). This pattern of the effective reproduction number identified with our approach is similar to the one computed using dynamical system model^33^. The maintenance of the effective reproduction number below 1 observed in our study due to the policies that control imported cases is consistent with the estimated low reproduction number in other places where inter-cities travel restrictions were imposed^36,39,41^. Despite a continuous flow of cases from India, one of the most COVID-19 affected countries in the world, the policies implemented by the Nepal government ideally controlled the local transmission, and thus deserve in-depth analysis to identify their local level impacts. The COVID-19 epidemic control through travel restrictions and non-pharmaceutical interventions have also been reported in other places^4,12,14,17,35–37,39–43^.

More importantly, our results show that the magnitude and trend of the epidemic indicators widely vary among provinces, most likely due to heterogeneity of border-sharing with India and population contact network. The magnitude and timing of peaks, as well as the slope of the linear trend (at the beginning of each surge), is quite different from province to province (Table 1). The province-wise variation is clearly seen in the pattern of daily growth rate as well (Fig. 1). For example, Province 3, which does not share a border check-point with India, has a negative growth rate during the early period of the pandemic, as opposed to other provinces (2, 5, 6, 7), which experience high mobility of migrant workers across open borders. Disparities in the growth rate has also been reported in a study that examined the effects of human mobility and control measures in provinces of China^12^. The effective reproduction number, which is widely considered to observe disease trends impacted by policies, was also found to vary widely among provinces both in magnitude as well as timing to cross threshold value of 1 (Fig. 4). In line with our finding, the disparity in $R_{t}$-trend has also been found in two cities of China where imported cases also contributed to the COVID-19 cases at the beginning of the epidemic^36^. Such wide disparities across provinces found by our study underscores that the analysis of country-level data might not be enough to implement policies for reducing province-level epidemic indicators.

Given the potential disparities of indicators among provinces, we further performed analysis through modeling to identify how the national-level policies implemented by the Nepal government were translated into province-level control. Our model comparison found that all four policies, lockdown, testing, isolation, quarantine, significantly contributed to each of the reported cases, the daily growth rate, and the effective reproduction number when the analysis is performed at the country level. However, the local level modeling indicates that the number and type of policy that was effective significantly vary among provinces as well as among epidemic indicators; only one policy (lockdown) was effective to reduce reproduction number for Province 4, while a combination of all four policies was effective for Province 1 and 7 (Table 3). On the other hand, for the same Province 4, three policies (testing, isolation, quarantine) were effective to alter the reported cases, while a different combination of three policies (lockdown, isolation, quarantine) was effective to alter the daily growth rate. In addition to the number and type of policies, the magnitude of effects of these policies is quite different from province to province (Table 4). In general, lockdown appeared to be the most effective policy to control most of the indicators, consistent with previous studies^33,36,37,39,40,42,43^, although there were some exceptions. Combining all these analyses, our results indicate that the net effect of the policies can be province-dependent despite the policy planning at national level, and thus province-level analysis is critical to proper implementation of government policies.

We acknowledge several limitations of our study. The data used in this study was limited and bears some uncertainties. In particular, the COVID-19 cases include only those that were tested and confirmed positive. However, several studies^6,18,58^ have documented asymptomatic or undiagnosed COVID-19 infected individuals who can spread the virus, even though their infectivity still remains uncertain. Therefore, our estimates may have underestimated the values of indicators, especially the reproduction numbers. The actual number of active cases was not available, thus there is some uncertainty on the computed daily growth rates. Despite the strict policy of the government of Nepal to perform border screens, there were cases that avoided the border screening, causing potential direct community transmission by infected migrants. These cases, while assumed to be negligible, may contribute to the uncertainty of our estimates of growth rate and reproduction number. In our modeling, the effects of policies were assumed to be linear. However, the infectious disease transmission mechanism, especially in the presence of multiple policies, can be nonlinear. We note that our models provided positive coefficients in some cases, showing the increase in policy level in those cases was associated with the increased epidemic indicator. As discussed earlier, while it appears to be paradoxical, this may be possible to be true in the context of Nepal. For example, higher testing and isolation can be associated with a higher reproduction number because the testing and isolation were primarily focused on seasonal migrants rather than individuals in the local community. It is worth mentioning that we obtained all coefficients negatives when we restricted our model to a single predictor. Uncertainty around these results needs more accurate data along with complex nonlinear models, such as dynamical system models.

In summary, our study on province-wise COVID-19 data from Nepal provides a baseline for evaluating local-level epidemic indicators under the commonly practiced national-level policy implementation. As demonstrated by our analyses, the net effects of nationally implemented policies can widely vary on the province-level epidemic indicators due to heterogeneity on the geographical situation (open border with India) and the contact network (population size and urbanization). Therefore, local level analyses are important for estimating epidemic indicators and designing ideal non-pharmaceutical policies for the control of local COVID-19 outbreaks.

## Methods

### Data collection and analysis

The countrywide and province-wise data of COVID-19 outbreak and the government policies implemented were obtained from a wide range of publicly available sources, including official websites of different ministries of Nepal, public health information sources, and national and regional newspapers^30,44,59–61^. Specifically, the numerical data include “reported case number”, “recovered case number”, “death case number”, “number of tests performed”, “number of people sent to quarantine and isolation” for each province as well as the whole country. In this study, we considered the number of new cases and active cases data from March 29 through October 15, 2020 and the available policy-related data from March 29 through September 27, 2020. Since the active case numbers were not available, we approximated them by subtracting the recovered case number and death number from the reported case number. A few missing data are estimated by taking the average between the value from the previous day and the following day.

As suggested by several government agencies, most of the new cases before mid-June were seen in the people who recently returned from India^62^. Thus we assumed that 80% of the new incidences before June 18, 2020 were contributed by imported cases, and after June 18, 2020, all the new cases were assumed to be local cases. We also considered the data set generated assuming 70% and 90% contributed by the imported cases and found that the results with these new data sets are similar to the ones with 80% imported cases. Therefore, all the results presented in this study are based on 80% of cases contributed by imported cases before June 18, 2020. All the compiled data sets that we used in this study are provided in the GitHub public repository (see Data Availability).

### Estimation of the epidemic growth rate

The daily exponential growth rate has been considered to be a useful indicator of epidemic progress. As discussed in Hsiang et al.^34^, the number of infected cases for a short time period ($t_{1}$ to $t_{2}$) can be approximated by an exponential growth model. Thus, we take

$$\begin{aligned} I_{t_{2}}=I_{t_{1}}e^{g_{t_{1}}(t_{2}-t_{1})}. \end{aligned}$$

Taking the natural logarithm on both sides we get $\ln {(I_{t_{2}})}-\ln {(I_{t_{1}})}=g_{t_{1}}(t_{2}-t_{1})$. For the daily growth rate, we take $t_{2}-t_{1}=1$, which implies the daily growth rate at time $t_{1}$ to be

$$\begin{aligned} g_{t_{1}}=\ln {(I_{t_{2}})}-\ln {(I_{t1})}. \end{aligned}$$

### Estimation of the basic reproduction number, $R_{0}$

The basic reproduction number, $R_{0}$, is defined as the number of new infections produced by an infected individual during its infection lifetime^63^. There is a number of methods to estimate the basic reproduction number from the disease incidence data. Among them, the method proposed by White and Pagano^56,64^, known as the maximum likelihood method (MLM), is widely used in many studies. Therefore, we selected MLM to estimate $R_{0}$ for Nepal and its provinces. However, for the purpose of comparison, we also estimated $R_{0}$ using the exponential growth method (EGM), and obtained the values for $R_{0}$ similar to the ones obtained by MLM. To implement MLM, we require the incidence data, the number of new cases over consecutive time units represented by $(N_{0},N_{1},\ldots ,N_{T})$, and the distribution of generation time, which is defined as the time duration from onset of infectiousness in a primary case to the onset of infectiousness in a secondary case that was infected by the primary case^65^. We note that at each time *t*, the total incident cases, $N_{t}$, is the sum of the number of incident local cases ($N_{t}^{\text {local}}$) and imported cases ($N_{t}^{\text {imported}}$), i.e., $N_{t} = N_{t}^{\text {local}}+N_{t}^{\text {imported}}$.

Since the generation time is a nonobservable phenomenon, especially in COVID-19 due to the presence of incubation phase, we approximated it by the serial interval, which is defined as the duration of time between the onset of symptoms in a primary case (infector) and the onset of symptoms in a secondary case (infectee) that was infected by the primary case^56,65^. Following the previous studies^38,66–68^, in which the data on the serial interval of COVID-19 was found to follow the gamma distribution, we used the gamma-distribution for the serial interval in our computation. In this study, we present the results with the gamma distributed serial interval with the mean of 3.96 days and the standard deviation of 4.75 days as reported by Du et al.^69^, while we note that some other studies have reported the mean varying from 1.77 to 5.30^38,66–68^.

We assumed that the secondary cases produced by an infected individual follow a Poisson process, with an expected value $R_{0}$. If $w_{s}$ is a distribution of the serial interval, $R_{0}$ can be estimated by maximizing the log-likelihood function

$$\begin{aligned} LL(R_{0})=\sum _{t=1}^{T}\ln \left( \frac{e^{-\mu _{t}}\mu _{t}^{N_{t}^{\text {local}}}}{N_{t}^{\text {local}} !}\right) \end{aligned}$$

where $\mu _{t}=R_{0}\sum _{s=1}^{t}N_{t-s}w_{s}$. This provides the following expression for $R_{0}$

$$\begin{aligned} R_{0}=\frac{\sum _{t=1}^{T} N_{t}^{\text {local}}}{\sum _{t=1}^{T}\sum _{s=1}^{t}N_{t-s}w_{s}}. \end{aligned}$$

We carried out the computation in R-software using ‘$R_{0}$’ package that implements MLM to estimate the basic reproduction number for Nepal and its provinces.

### Estimation of the effective reproduction number, $R_{t}$

The effective reproduction number, $R_{t}$, is the real-time estimation of the reproduction number. We use a similar idea as in the case of $R_{0}$ and assume poisson process^56,57,70^. Therefore for the time-dependent $R_{t}$, the likelihood function becomes

$$\begin{aligned} L(R_{t,\tau })=\prod _{k=t-\tau }^{t} \frac{e^{-R_{t,\tau }\Psi _{k}}(R_{t,\tau }\Psi _{k})^{N_{k}^{\text {local}}}}{N_{k}^{\text {local}}!} \end{aligned}$$

where, $\Psi _{k}=\sum _{s=1}^{k}N_{k-s}w_{s}$. Similar to the technique implemented in previous studies^57,70^, $\tau$ represents the length of the time window over which $R_{t}$ is assumed to be constant. In the Bayesian framework, it can be shown that a Gamma prior distribution for $R_{t,\tau }$ with parameters (*a*, *b*) implies the posterior distribution of $R_{t,\tau }$ to be Gamma distribution with parameters^57,70^

$$\begin{aligned} \left( a+ \sum _{k=t-\tau }^{t}N_{k}^{\text {local}},~~\frac{1}{\frac{1}{b}+\sum _{k=t-\tau }^{t}\sum _{s=1}^{k}N_{k-s}w_{s}}\right) . \end{aligned}$$

As in the calculation of $R_{0}$, we consider the serial interval following gamma distribution with the mean of 3.96 days and the standard deviation of 4.75 days^69^. All computations were performed in R-software using ‘*EpiEstim*’ package^57,70^.

### Modeling via multiple regression analysis

We considered four policies implemented by the government of Nepal: lockdown ($L_{t}$), testing ($T_{t}$), isolation ($I_{t}$), and quarantine ($Q_{t}$). Assuming the linear relationship between these policies and the epidemic indicators (the reported case number, $N_{t}$, the daily growth rate, $g_{t}$, and the effective reproduction number, $R_{t}$), we modeled each of the epidemic indicators using the multiple linear regression as follows^34,71,72^

$$\begin{aligned} y_{t}=\beta _{0}+\beta _{L} \cdot L_{t}+\beta _{T} \cdot T_{t}+\beta _{I} \cdot I_{t}+\beta _{Q} \cdot Q_{t}+ \varepsilon _{t}, \end{aligned}$$

where $y_{t}$ represents one of the three epidemic indicators ($N_{t}$, $g_{t}$, $R_{t}$), whose analysis is required. Here, $\beta _{0}$, $\beta _{L}$, $\beta _{T}$, $\beta _{I}$, and $\beta _{Q}$ represent regression coefficients corresponding to no policy, lockdown, testing, isolation, and quarantine, respectively. $\varepsilon _{t}$ is the residual term, which is assumed to have an expected value of zero with constant variance and to be independently and identically distributed. As per the available data, the lockdown is a categorical variable (taken as $L_{t} = 1$ or 0 depending on whether or not lockdown is in effect), and the remaining other three policies are numerical.

For each epidemic indicator, we considered all possible models with 1, 2, 3, 4 policies taken at a time, i.e., the total of 15 possible models. We performed data fitting to these models, and chose the best model on the basis of their AIC (Akaike information criterion) values, which is given by^71,72^

$$ {\text{AIC}} = \frac{1}{{n\widehat{\sigma }^{2} }}\left( {{\text{RSS}} + 2d\widehat{\sigma }^{2} } \right). $$

Here *n* is the number of days for which the data was recorded, ${\hat{\sigma }}^{2}$ is an estimate of the variance of the residual $\varepsilon _{t}$, RSS is the residual sum of squares, and *d* is the number of policies in the model. We repeated the process for each province and for the whole country.

## Data Availability

The compiled data that are analyzed are available in the github public repository. https://github.com/Subas-Acharya/COVID_Project.
